# Systematic review of the method and quality of reporting of complications from studies evaluating innovative glaucoma surgical procedures

**DOI:** 10.1038/s41433-022-02268-z

**Published:** 2022-10-17

**Authors:** Jonathan Bonnar, Augusto Azuara-Blanco

**Affiliations:** 1 Belfast Health and Social Care Trust, Belfast, UK; 2 Queen’s University Belfast, Belfast, UK

**Keywords:** Surgery, Adverse effects

## Abstract

The objective of this systematic review is to identify how reporting of micro-invasive glaucoma surgery (MIGS) procedure complications are reported in randomised controlled trials (RCTs) and the quality of this reporting compared to the CONSORT extension for harms. RCTs evaluating MIGS procedures were identified from a database of systematic reviews and from recent literature. Trials were evaluated in comparison to the CONSORT extension for harms to quantify the quality of harms reporting. Simple descriptive statistics were calculated for the CONSORT checklist. 21 trials were identified as eligible for inclusion, 14 were evaluating iStent, one Trabectome, three Hydrus, one Cypass, one Preseflo MicroShunt and one Excimer laser trabeculotomy. The average number of CONSORT for Harms checklist items fulfilled by the studies was 10 out of 16. No studies used a validated instrument to report severity of harms and only 4 had a list or definition of adverse events. An analysis of harm was conducted by 19 of 21 studies (90%). Appropriate metrics were used for reporting rates of adverse events in 19 of 21 studies but in only 4 studies was there an attempt to give these adverse events a grade of seriousness. In conclusion, most studies evaluating MIGS procedures do make an effort to acknowledge harms data, however this is not done uniformly well or in the same manner. A validated instrument to report severity and a standard list of complications for MIGS surgery would go a long way to helping this.

## Abstract (zh)

本系统综述的目的是发现在随机对照试验 (RCT) 中如何报告微创青光眼手术 (MIGS) 的并发症, 以及对比该报告与随机对照临床试验报告规范 (CONSORT) 损害扩展声明的质量。评估MIGS的RCT来自于最近文献及系统性综述数据库。与CONSORT损害扩展声明相比, 我们对试验进行了评估, 以量化损害报告的质量。用CONSORT检查表计算简单的描述性统计。21项临床试验符合纳入条件, 其中14项临床研究评估了iStent、一项小梁消融术、三项Hydrus微型支架、一项CyPass微型支架、一项Preseflo MicroShunt (一种青光眼引流器) 和一项准分子激光小梁切开术。本研究完成的CONSORT损害检查表的平均数量为10/16。没有研究使用经验证的工具报告损害的严重程度, 只有4项研究有不良事件的列表或定义。21项研究中的19项 (90%) 进行了损害分析。21项研究中的19项 (90%) 进行了危害分析。21项研究中有19项使用了适当的指标来报告不良事件的发生率, 但只有4项研究试图对这些不良事件的严重性进行分级。总之, 大多数评估MIGS程序的研究尽量引用了损害数据, 但这并不是以统一或相同的方式完成的。一个验证性的工具报告MIGS手术的严重程度和标准并发症的列表将会对其有长期的帮助。

## Introduction

Over the past decade there has been a paradigm shift in the world of glaucoma surgery from traditional procedures such as the trabeculectomy and tube drainage devices to a wide range of new techniques and devices which purport to be able to lower intraocular pressure (IOP) in a less invasive manner. These are collectively called micro-invasive glaucoma surgery (MIGS), although there is no widely accepted definition of what can and cannot be referred to by this term. One of the key tenets of some of the novel procedures is that although they may not lower IOP as much as traditional surgery, they are safer [1], particularly surgeries not associated with a filtering bleb. This has led to a change in when glaucoma surgery is performed as MIGS may be used to reduce drop burden as an add-on to cataract surgery, to improve quality of life for our patients or in mild to moderate disease. This may mean that patients are undergoing ‘glaucoma surgery’ at a much earlier stage in their disease journey, with some studies even performing MIGS at diagnosis [2]. We must therefore ensure that these techniques and devices are rigorously tested for safety and efficacy in order to be able to recommend them, with full confidence that they are the best option for our patients.

In 2019, the World Glaucoma Association published consensus guidelines on the design and reporting of glaucoma surgical trials and included in this, guidelines on the reporting of complications [3]. This has a list of standardised definitions of complications, and tables to report their occurrence. There are tables for complications related to trabeculectomy, drainage devices and non-penetrating glaucoma surgeries, but none relating specifically to MIGS.

Adequate reporting and quantification of severity of complications is an important consideration when evaluating surgical innovations. Sii et al. highlighted deficiencies in the reporting of complications in glaucoma surgical trials [4]. This review identified trials published before 2017, but since them a number of trials evaluating MIGS have been reported.

In this study, we identified how complications were reported and the quality of the reporting in MIGS trials.

## Methods

We identified systematic reviews and randomised controlled trials (RCTs) on surgical interventions for glaucoma. The protocol for this review has been registered in the online database PROSPERO (CRD42021278766).

The Cochrane Eyes and Vision United States Satellite maintains a database of Cochrane and non-Cochrane systematic reviews and meta-analyses in vision research and eye care. The full search strategy for this database has been published elsewhere [5]. We complemented this strategy with a systematic search of RCTs of the last 5 years, from January 1, 2016, to June 16, 2021. The electronic databases Cochrane Library, Medline, Embase, Scopus and Clinical Trials.gov were used. Searches were conducted by one investigator and validated by a senior investigator. This efficient methodology has been validated proving that systematic reviews may not need to conduct independent dual abstraction [6].

The population of interest was adult patients with glaucoma of any type, with or without co-existing cataract. As intervention, we considered any novel glaucoma surgery, including MIGS procedures performed for any reason, either alone or in combination with cataract surgery. We excluded studies evaluating outcomes of traditional glaucoma surgery (i.e. trabeculectomy, or modifications of trabeculectomy such as Ex-Press shunt, glaucoma drainage device insertion), interventions for congenital glaucoma, and laser therapy.

As comparator we included any control or alternative intervention.

For each trial identified data on the reporting of complications was extracted by one investigator and checked by a second investigator against the CONSORT extension for harms criteria [7]. We did not re-extract data or re-assess the risk of bias of the individual studies in the reviews.

The CONSORT extension for harms contains ten recommendations about reporting harms-related issues. Some of these are quite broad and so were subdivided to enhance the quality of data collection and its ease (Table 1) [8]. Each item was marked as 0 (No) or 1 (Yes). If a trial has a published study protocol, this was also accessed to review for additional information. Any disagreement by reviewing authors was agreed by discussion to ensure consistency across all studies. Simple descriptive statistics were calculated for the number of checklist items completed for each study and for the number of studies completing each checklist item.

**Table 1:** Description of each component of the CONSORT Extension for Harms and how it was applied in studies.

1) Title/Abstract	Are adverse events or complications mentioned in title or abstract?
2) Introduction	Are adverse events or complications mentioned in the introduction?
3) Definition of adverse event	
a. List or definition b. Expected v unexpected c. Validated instrument to report severity	a. Was a list or definition of adverse events produced prior to the study starting? b. Are expected and unexpected adverse events addressed prior to study starting? c. Is a validated instrument used to report severity?
4) Collection of harms data	
a. Mode of collection b. Timing of collection c. Attribution methods d. Monitoring and stopping rules	a. Is there a description of how harms data is collected? b. Is the timing of harms data collection made clear? c. Is there a process described for apportioning the harm incurred to the intervention being studied? d. Are rules in place prior to the study commencing which would stop the study early if harms are resulting?
5) Analysis of harm	Is an analysis of adverse events produced? Simple descriptive statistics is considered adequate.
6) Participant withdrawals	
a. Withdrawals due to harm and experiences b. Timing	a. Are withdrawals from the study accounted for with a reason given for withdrawal? b. Is the timing of withdrawals reported? (Studies which reported no withdrawals were awarded yes for both if withdrawals were explicitly addressed)
7) Denominators for analyses of harm	
a. Denominators for adverse events b. Definitions used for analysis	a. Is it clear how many patients are being considered as having undergone treatment in harms analysis? b. Is the type of analysis being performed explained?
8) Data on adverse events	
a. Appropriate metrics b. Grade or seriousness	a. Are harms appropriately presented? b. Any effort made to present the seriousness of adverse events.
9) Subgroup analyses for harm	Are subgroups analysed for harms?
10) Balanced discussion	Does the discussion address harms appropriately?

## Results

**Fig. 1:**
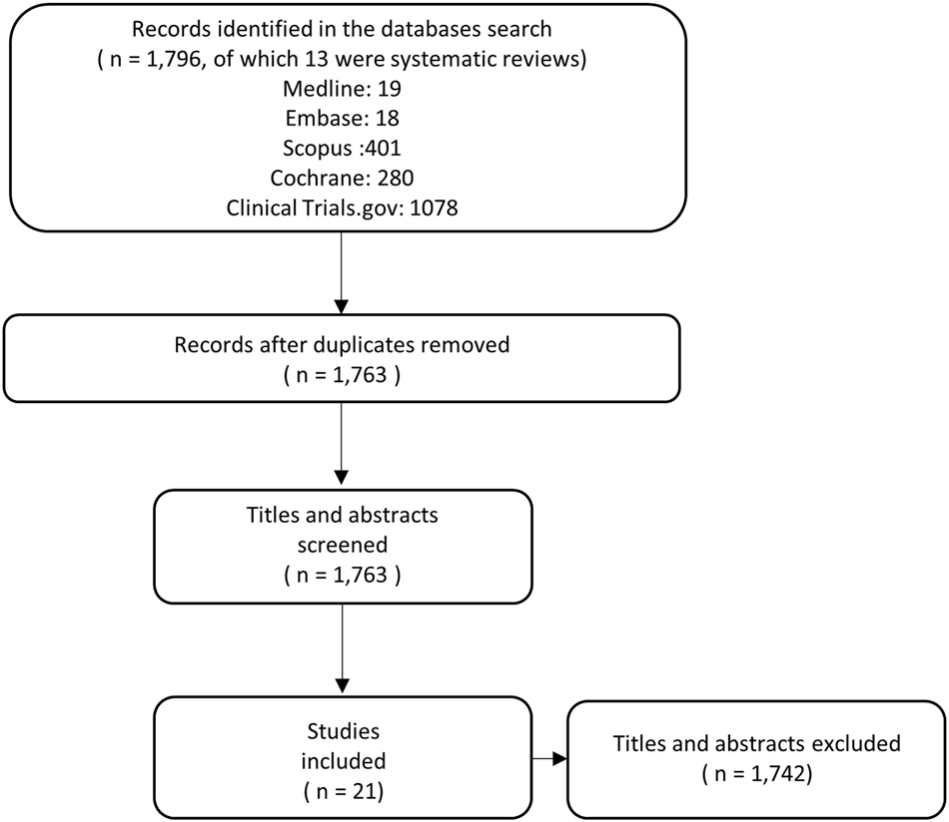
PRISMA flow diagram of study selection. Diagram shows records identified by search, then screening and inclusion or exclusion.

The PRISMA flowchart (Fig. 1) and a list of studies identified in the databases, including 13 systematic reviews on glaucoma.

A total of 21 trials were identified as eligible for inclusion. Of the 21 included studies, 14 were evaluating iStent, one Trabectome, three Hydrus, one Cypass, one Preseflo MicroShunt and one Excimer laser trabeculotomy outcomes (Table 2).

**Table 2:** Included RCTs.

Author	Year	MIGS
Babighian [11]	2010	ELT
Fea [12]	2010	iStent/Phaco
Fernandez-Barrientos [13]	2010	iStent/Phaco
Samuelson [14]	2011	iStent/Phaco
Craven [15]	2012	iStent/Phaco
Fea [16]	2014	iStent
Fea [17]	2015	iStent/Phaco
Katz [18]	2015	iStent
Pfeiffer [19]	2015	Hydrus/Phaco
Vold [20]	2016	iStent
Vold [21]	2016	Cypass/Phaco
Arimura [22]	2018	Ex-PRESS
Katz [23]	2018	iStent
Ting [24]	2018	Trabectome
Ahmed [25]	2019	Hydrus v iStent
Samuelson [26]	2019	Hydrus/Phaco
Samuelson [27]	2019	iStent
Chen [28]	2020	iStent
Dorairaj [29]	2020	iStent/KDB
Falkenberry [30]	2020	iStent/KDB
Baker [31]	2021	MicroShunt

**Fig. 2:**
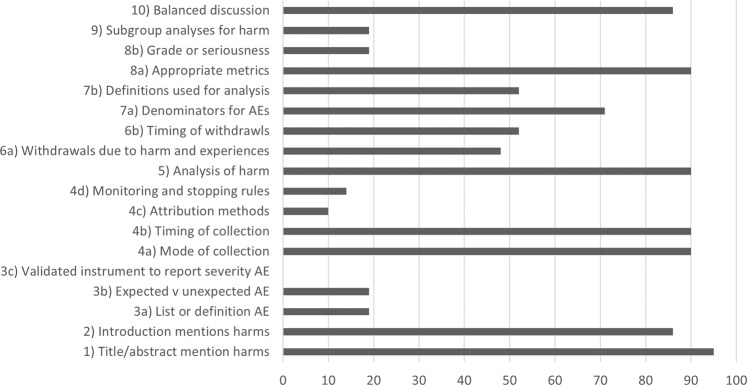
Percentage of studies completing CONSORT for harms checklist item. Graph shows each CONSORT for harms checklist item and the percentage of included studies which fulfilled each item.

The average number of CONSORT for Harms checklist items fulfilled by the studies was 10 out of 16 (63%, range 2–15). No studies used a validated instrument to report severity, only 4 had a list or definition of adverse events and only 4 differentiated between expected and unexpected events (Fig. 2). The mode and timing of collection of harms data was well reported with 19 of 21 reporting this (90%). However attribution methods and monitoring and stopping rules were poorly recorded at 2 and 3 out of 21 respectively. An analysis of harm was conducted by 19 of 21 studies (90%). Participant withdrawals were recorded in 10 of 21 studies however the quality of this reporting varied and it was not always apparent why the participants had withdrawn or when. Appropriate metrics were used for reporting rates of adverse events in 19 of 21 studies but in only 4 studies was there an attempt to give these adverse events a grade of seriousness. Subgroup analysis on harms was carried out in 4 of 21 studies and 18 of 21 were felt to provide a balanced discussion of the harms of the intervention.

## Discussion

With the introduction of novel surgical techniques an evaluation of efficacy and harms is essential for surgeons and patients to be able to compare and choose among different procedures.

As we think back to the key principles of medicine, the concept of ‘primum non nocere’ or ‘first do no harm’ is a cornerstone of modern practice. We must therefore acknowledge that reporting of surgical complications is one of the most important metrics when evaluating a new surgical technique or device for use. The CONSORT extension for harms was designed to help reporting this important domain in a thorough and structured manner. From our review, we can see that most studies are attempting to consider harms related data as part of their approach. It is apparent however that some factors are not present which would aid this. No studies used a validated instrument to report severity and very few used a list or definitions. We feel that these would assist greatly in the study of harms related data in glaucoma surgical trials of MIGS surgery. Stringa et al. have recently published a list of complications of glaucoma surgery [9]. This comprehensive review looked at the naming of complications and their definitions across multiple studies, combining similar complications and producing definitions of each based on expert opinion. This will allow future studies to use the same definitions, hence allowing comparison across different glaucoma surgical techniques.

It is also important to acknowledge that RCTs may not be able to identify uncommon complications due to their sample size, with very large RCTs powered to detect these complications being too large and expensive to run. Registries where surgeons report clinical outcomes and complications or real world data are better suited to detect less frequent complications of surgical interventions.

Our study does have some limitations, there are not yet many RCTs evaluating harms in MIGS surgery and so we could only include a relatively small number of studies. Many MIGS techniques are also quite new and so longer term data regarding safety has yet to be established. The strengths however are the systematic search and also the use of the CONSORT extension for harms which is a well established method of reporting harms in RCTs. This study complements the recent overview on MIGS devices and highlights that we have no robust evidence to be able to compare effectiveness and safety among different devices [10].
